# The Prevalence and Risk Factors for Severe Maternal Morbidities: A Systematic Review and Meta-Analysis

**DOI:** 10.3389/fmed.2022.861028

**Published:** 2022-03-17

**Authors:** Nik Hussain Nik Hazlina, Mohd Noor Norhayati, Ismail Shaiful Bahari, Halilul Rahman Mohamed Kamil

**Affiliations:** ^1^Women's Health Development Unit, School of Medical Sciences, Universiti Sains Malaysia, Kubang Kerian, Malaysia; ^2^Department of Family Medicine, School of Medical Sciences, Universiti Sains Malaysia, Kubang Kerian, Malaysia

**Keywords:** severe maternal morbidity, maternal near miss, prevalence, risk factor, meta-analysis

## Abstract

**Introduction:**

Maternal mortality and severe maternal morbidity remain major public health problems globally. Understanding their risk factors may result in better treatment solutions and preventive measures for maternal health. This review aims to identify the prevalence and risk factors of severe maternal morbidity (SMM) and maternal near miss (MNM).

**Methods:**

A systematic review and meta-analysis was conducted to assess the prevalence and risk factors of SMM and MNM. The study adhered to the Preferred Reporting Items for Systematic Reviews and Meta-Analysis guidelines. A systematic search was performed in the MEDLINE (PubMed), CINAHL (EBSCOhost), and Science Direct databases for articles published between 2011 and 2020.

**Results:**

Twenty-four of the 44 studies included were assessed as being of good quality and having a low risk of bias. The prevalence of SMM and MNM was 2.45% (95% CI: 2.03, 2.88) and 1.68% (95% CI: 1.42, 1.95), respectively. The risk factors for SMM included history of cesarean section (OR [95% CI]: 1.63 [1.43, 1.87]), young maternal age (OR [95% CI]: 0.71 [0.60, 0.83]), singleton pregnancy (OR [95% CI]: 0.42 [0.32, 0.55]), vaginal delivery (OR [95% CI]: 0.11 [0.02, 0.47]), coexisting medical conditions (OR [95% CI]: 1.51 [1.28, 1.78]), and preterm gestation (OR [95% CI]: 0.14 [0.08, 0.23]). The sole risk factor for MNM was a history of cesarean section (OR [95% CI]: 2.68 [1.41, 5.10]).

**Conclusions:**

Maternal age, coexisting medical conditions, history of abortion and cesarean delivery, gestational age, parity, and mode of delivery are associated with SMM and MNM. This helps us better understand the risk factors and their strength of association with SMM and MNM. Thus, initiatives such as educational programs, campaigns, and early detection of risk factors are recommended. Proper follow-up is important to monitor the progression of maternal health during the antenatal and postnatal periods.

**Systematic Review Registration:**

https://www.crd.york.ac.uk/prospero/display_record.php?ID=CRD42021226137, identifier: CRD42021226137.

## Introduction

Severe maternal morbidity (SMM) refers to “potentially life-threatening conditions during pregnancy, childbirth or after pregnancy, from which maternal near miss (MNM) cases would emerge” (1, 2). The standard definition and internationally accepted identification criteria for SMM by the World Health Organization (WHO) Working Group on Maternal Mortality Morbidity Classifications (1, 2) incorporate clinical disorders (hemorrhagic, hypertensive, and other systemic disorders) and severe management indicators to assess the severity of SMM and to strengthen the ability of health care professionals to detect SMM (1).

However, maternal mortality and SMM remain major public health problems for global healthcare systems despite substantial progress (3). Between 1990 and 2015, the global maternal mortality ratio (MMR) declined by 44% but failed to meet the 75% reduction set by Millennium Development Goal 5. Sustainable Development Goal 3 aims to ensure healthy living, promote well-being for people of all ages, and reduce global MMR to <70 per 100,000 live births by 2030. The average reduction of 7.5% each year between 2016 and 2030 is more than three times the 2.3% annual rate of decline observed globally between 1990 and 2015.

Prior to 2011, the literature varied in its definitions and criteria used to classify SMM and MNM cases. In a review, the prevalence of SMM based on various disease-specific, management-based, and organ system dysfunction-based criteria ranged from 0.04 to 15% (4). Meanwhile, the prevalence according to organ-based dysfunction using the Mantel criteria and emergency hysterectomy was 0.42 and 0.039%, respectively. With a 98.3% *I*^2^ value for the Mantel-based criteria and 95.5% for the emergency hysterectomy criteria, the heterogeneity among studies was large and significant (4).

While maternal mortality has been commonly used as a benchmark for maternal health status, there is evidence that it is just the “tip of the iceberg” for adverse maternal outcomes (5, 6). The SMM literature has revealed several contributing factors. Employment status (7), low household income (8), history of abortion (8), multiple births (9), and inadequate antenatal care (8) were documented as contributing factors for SMM. However, mixed findings were reported for the factors of age (10–12), race (9, 13), educational level (8, 13), co-existing medical conditions (14), parity (15), gestational period (10), mode of delivery (12, 16), previous cesarean section (14), and pre-pregnancy body mass index (9, 14).

Determining the pooled prevalence of SMM at a global level gives a better indication of its severity than discrete primary studies. The identification of factors associated with SMM allows a clearer understanding of the issue and serves as a basis for appropriate preventive strategies to be established. This pertains to primary prevention at the institutional, provider, and client levels, such as screening or preventive that could potentially avoid death or severe morbidity from a disorder. Therefore, to inform clinical policy and enhance patient care, planning beyond maternal mortality and direct, focused evaluation of the strength of each risk factor's association with SMM is required. This systematic review aims to ascertain the prevalence and risk factors of SMM based on the WHO standard definition and criteria (1). The evidence, effect estimates, and strength of statistical association between SMM and its risk factors will be summarized in this review.

## Methods

### Study Design and Search Strategy

**Figure 1:**
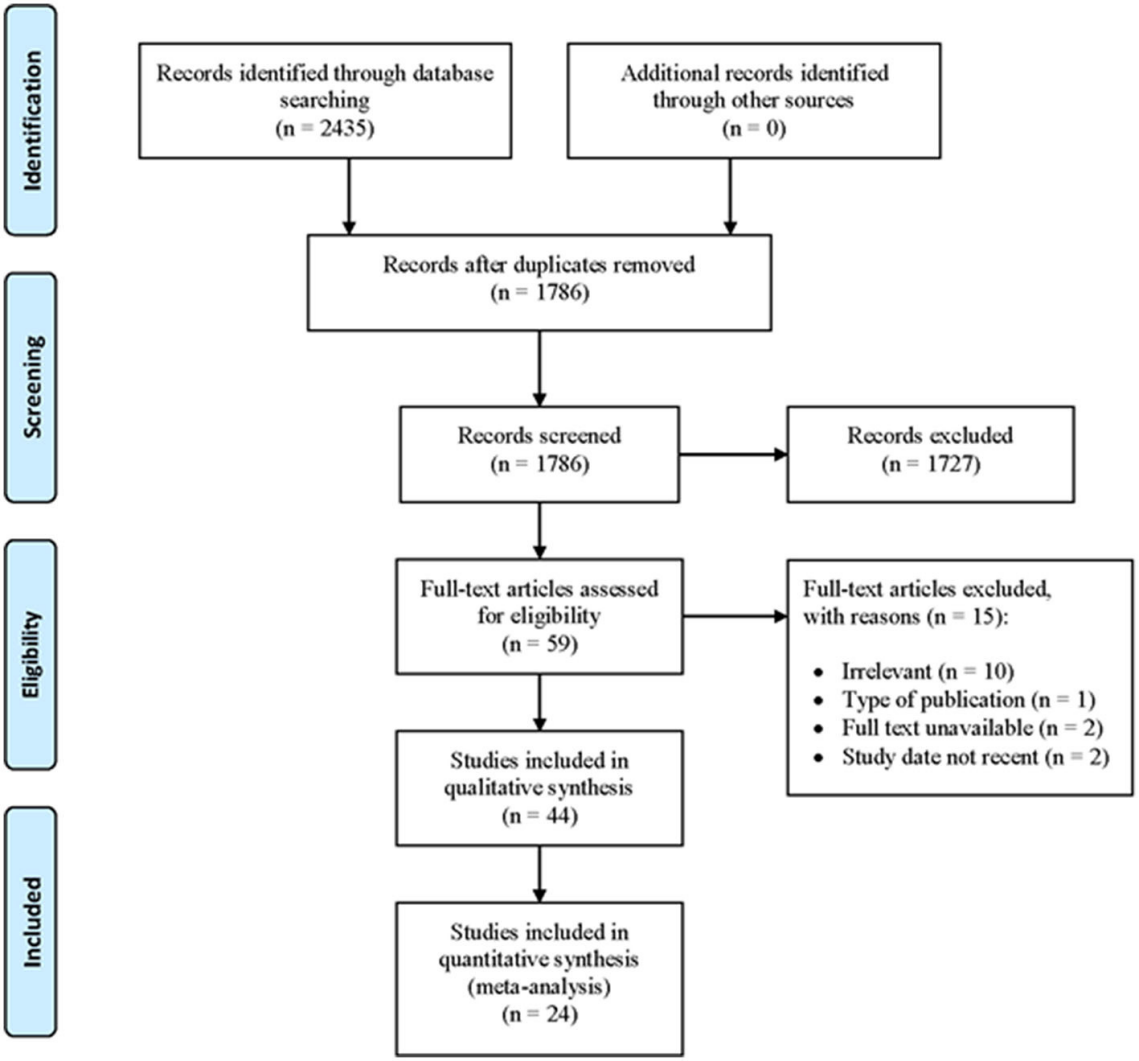
PRISMA flow chart.

To determine the prevalence and risk factors, researchers conducted a systematic review and meta-analysis of studies on SMM. The study followed the Preferred Reporting Items for Systematic Reviews and Meta-Analyses (PRISMA) guidelines (Figure 1).

A systematic search was performed in the MEDLINE (PubMed), CINAHL (EBSCOhost), and Science Direct databases for articles published between 2011 and 2020 inclusive. The search was done using the following Medical Subject Headings (MeSH) and generic free-text search terms as follows: ((Prevalence [MeSH Terms]) OR (risk factors[MeSH Terms])) AND (severe maternal morbidity[Text Word])) OR (maternal near miss[Text Word])) OR (potentially life-threatening[Text Word])) OR (maternal complications[Text Word])) OR (severe acute maternal morbidity[Text Word])) OR (pregnancy complications[Text Word])) AND ((“2011” [Date - Publication]: “2022” [Date - Publication])) The search terms were flexible and tailored to the various electronic databases. All studies published starting from 2011 were retrieved to assess their eligibility for inclusion in this study. The search was limited to full-text articles written in English. The reference lists of the included studies were cross-checked to locate additional potentially acceptable studies.

### Eligibility Criteria

The main inclusion criterion was the reporting of prevalence or risk factors of SMM. Studies that were written in English, published in 2011 or later, had cross-sectional, case-control, or cohort designs, and were conducted in the community or health institutions were included. Case series/reports, conference papers, proceedings, abstract-only articles, editorial reviews, letters of communications, commentaries, systematic reviews, and qualitative studies were excluded.

### Study Selection and Screening

All records identified using our search strategy were exported to the EndNote X8 (Clarivate Analytics). Duplicate articles were removed. Two independent reviewers (MKHR, NMN) screened the titles and abstracts of the identified articles. The full texts of eligible studies were obtained and thoroughly read to assess their suitability for the meta-analysis. A consensus discussion was held in the event of a conflict between the two reviewers, and a third reviewer was consulted. The search method is presented in the PRISMA flow chart showing the number of studies that were included and excluded, along with reasons for exclusion (Figure 1). The primary outcomes of this study were the prevalence and risk factors of SMM.

### Quality Assessment and Bias

A critical appraisal was done to assess data quality using the Joanna Briggs Institute Meta-Analysis for cross-sectional, case-control, and cohort studies (17). Independent bias assessments were carried out by two reviewers. When more than 70% of the responses were “yes,” moderate when 50–69% of the answers were “yes,” and high when <50% of the answers were “yes,” the risk of bias was rated low. Studies that showed a high or moderate risk of bias were excluded from the review.

### Data Extraction

Two reviewers independently extracted data into Microsoft Excel (Microsoft Office Professional Plus 2016). The data included the name of the first author, publication year, study location, study design, setting, study population, sample size, SMM definition, prevalence, risk factors, and data for calculation of effect estimates. The data for risk factors included sociodemographic characteristics, medical and gynecological histories, and past and present obstetric performance.

### Result Synthesis and Statistical Analysis

Outcomes were reported using odds ratios (ORs) and 95% confidence intervals (CIs). The analysis was performed with the Review Manager software (v.5.4; Nordic Cochrane Centre, Copenhagen, Denmark). A random-effects model was used to pool data. The *I*^2^ statistic was used to assess heterogeneity, which was stratified as follows: 0–40%, might not be important; 30–60%, moderate heterogeneity; 50–90% substantial heterogeneity; and 75–100%, considerable heterogeneity (18). Funnel plots and Egger's test were used to assess publication bias if indicated, and a *p* < 0.05 was declared as statistically significant.

## Results

### Characteristics of the Included Studies

A total of 2,435 articles were retrieved through an electronic search using the aforementioned search terms, of which 1,786 were eligible for the title and abstract assessment after removing 649 duplicate records. Of the 1,786 articles screened for eligibility, 1,727 were excluded after evaluating their titles and abstracts. A total of 59 articles underwent full-text assessment for eligibility, of which 15 were excluded due to irrelevancy (*n* = 10), not suitable type of publication (*n* = 1), unavailability of full text (*n* = 2), and not recent (*n* = 2) (Figure 1).

In this review, 44 articles underwent quality assessment, of which 24 with good quality and low risk of bias were included and the remaining poor-quality articles excluded. Nineteen studies were cross-sectional, two were case-control, and three were cohort. A variety of countries were represented in this systematic review and meta-analysis. The smallest sample size was 262 women, and the largest was 3,162,303 (Table 1). The source of funding of included articles was tabulated in the Supplementary File.

**Table 1:** Summary of research articles included in the systemic review and meta-analysis for SMM and MNM (*n* = 24).

**No**.	**References**	**Publication year**	**Study area (region)**	**Study design**	**Sample size**	**SMM**	**MNM**	**Prevalence of SMM (%)**
1	Lindquist et al. (19)	2015	Victoria, Australia	Case-control	211,060	1,119	N/A	0.53
2	Hitti et al. (20)	2018	University of Washington Medical Center	Cross-sectional	7,025	284	N/A	4.04
3	Dzakpasu et al. (21)	2019	Canada	Cross-sectional	1,418,545	22,799	N/A	1.61
4	Das et al. (22)	2014	Eastern India	Cross-sectional	6,100	99	N/A	1.62
5	Galvão et al. (23)	2020	Sergipe, Northern Brazil	Cross-sectional	16,243	1,102	77	6.78
6	Zhang et al. (24)	2020	Hebei, China	Cross-sectional	289,859	289,589	N/A	99.91
7	Norhayati et al. (12)	2016	Kelantan, Malaysia	Cross-sectional	23,422	352	N/A	1.50
8	Aoyama et al. (25)	2019	Canada	Cohort	3,162,303	54,219	N/A	1.72
9	Bashour et al. (26)	2015	Middle Eastern countries	Cross-sectional	9,063	N/A	71	0.78
10	Dessalegn et al. (27)	2020	Ethiopia	Case-control	321	0	80	24.92
11	Nansubuga et al. (28)	2013	Uganda	Cross-sectional	1,557	0	434	27.87
12	Rosendo et al. (29)	2017	Brazil	Cross-sectional	848	34	174	20.52
13	Chikadaya et al. (30)	2018	Zimbabwe	Cross-sectional	11,871	0	123	1.04
14	Rathod et al. (31)	2016	India	Cohort	21,992	0	161	0.73
15	Verschueren et al. (32)	2020	Suriname	Cohort	9,114	0	71	0.78
16	Iwuh et al. (33)	2014	Cape Town, South Africa	Cross-sectional	19,222	0	112	0.58
17	Dias et al. (34)	2014	Brazil	Cross-sectional	9,114	0	23,747	1.02
18	Domingues et al. (35)	2016	Brazil	Cross-sectional	19,222	0	243	1.02
19	Heemelaar et al. (36)	2020	Namibia	Cross-sectional	2,325,394	0	298	0.80
20	Mbachu et al. (37)	2017	Southern Nigeria	Cross-sectional	262	0	52	19.85
21	Ps et al. (38)	2013	Manipal University, India	Cross-sectional	7,390	0	131	1.77
22	Dile et al. (39)	2015	Ethiopia	Cross-sectional	806	0	188	23.33
23	Jayaratnam et al. (40)	2019	Timor Leste	Cross-sectional	4,702	0	39	0.83
24	Owolabi et al. (41)	2018	Kenya	Cross-sectional	182,571	0	1,278	0.70

*N/A, Not available; SMM, severe maternal morbidity; MNM, maternal near miss*.

### Severe Maternal Morbidity

**Figure 2:**
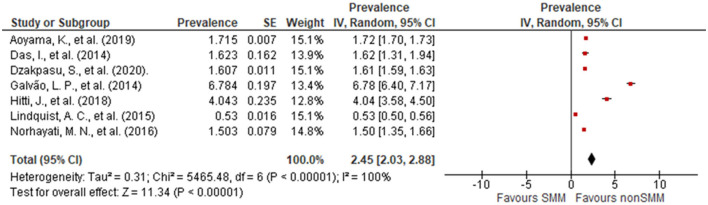
Forest plot depicting the prevalence of severe maternal morbidity.

We observed significant variations in the prevalence of SMM in this review (Figure 2). The lowest reported prevalence of SMM was 0.53% (19), and the highest was 6.78% (23). Eight articles were included, with the overall pooled prevalence of SMM at 2.45% (95% CI: 2.03, 2.88). Egger's test showed statistically insignificant publication bias (*P* = 0.162).

**Figure 3:**
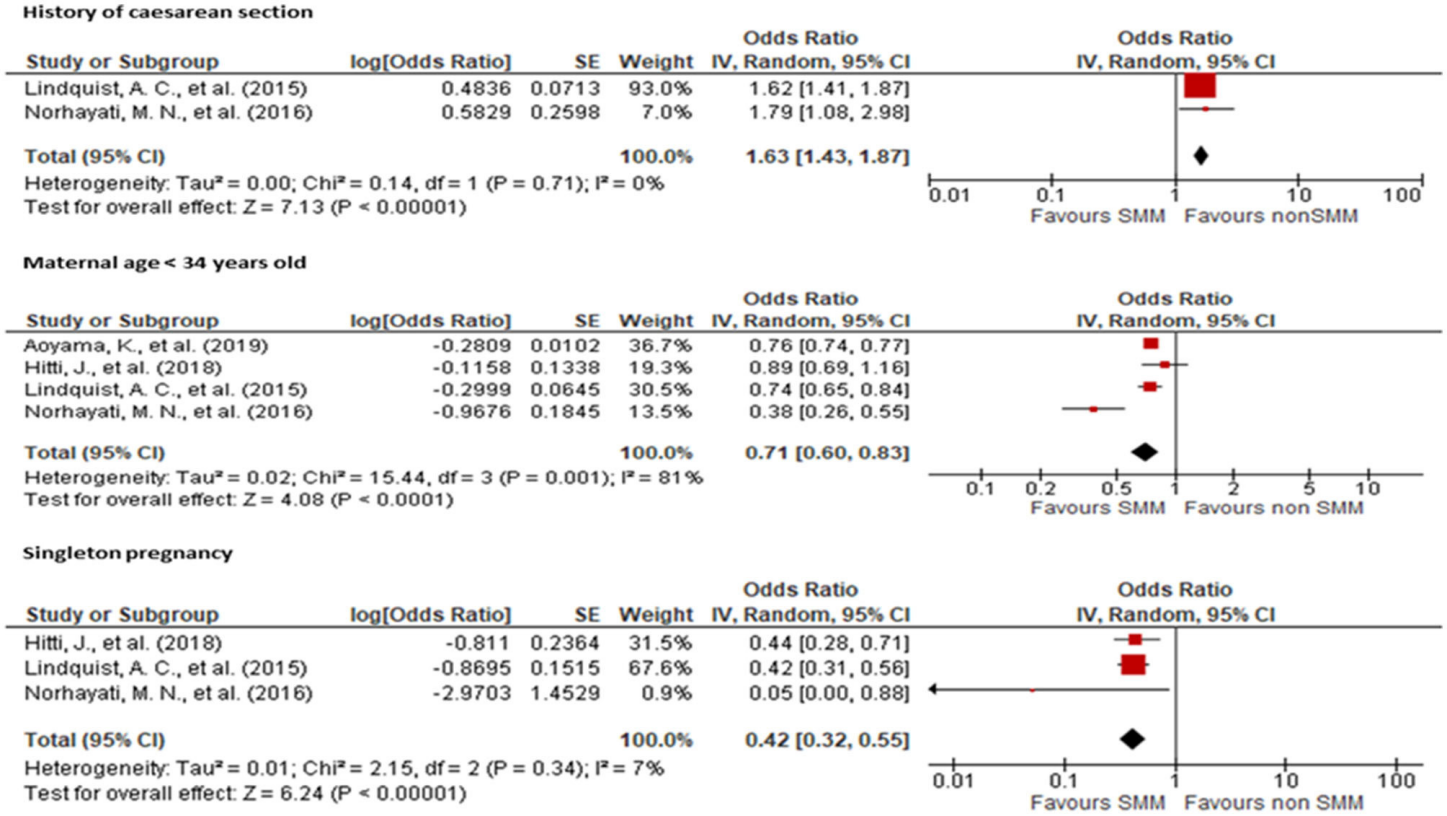
Forest plot showing the association of history of cesarean section, maternal age, and singleton pregnancy with severe maternal morbidity.

Various factors such as age, parity, history of cesarean section, previous history of abortion, singleton pregnancy, and coexisting medical conditions were evaluated for their association with SMM. Two studies (12, 19) reported an association between a history of cesarean section and SMM with an OR of 1.63 (95% CI: 1.43, 1.87) when compared to women with only a history of vaginal delivery. Four studies (12, 19, 20, 25) showed a significant association between young maternal age and SMM with an OR of 0.71 (95% CI: 0.60, 0.83) when compared with older women. Additionally, three studies (12, 19, 20) showed a significant association between singleton pregnancies and SMM with an OR of 0.42 (95% CI: 0.32, 0.55) when compared to twin pregnancies (Figure 3).

**Figure 4:**
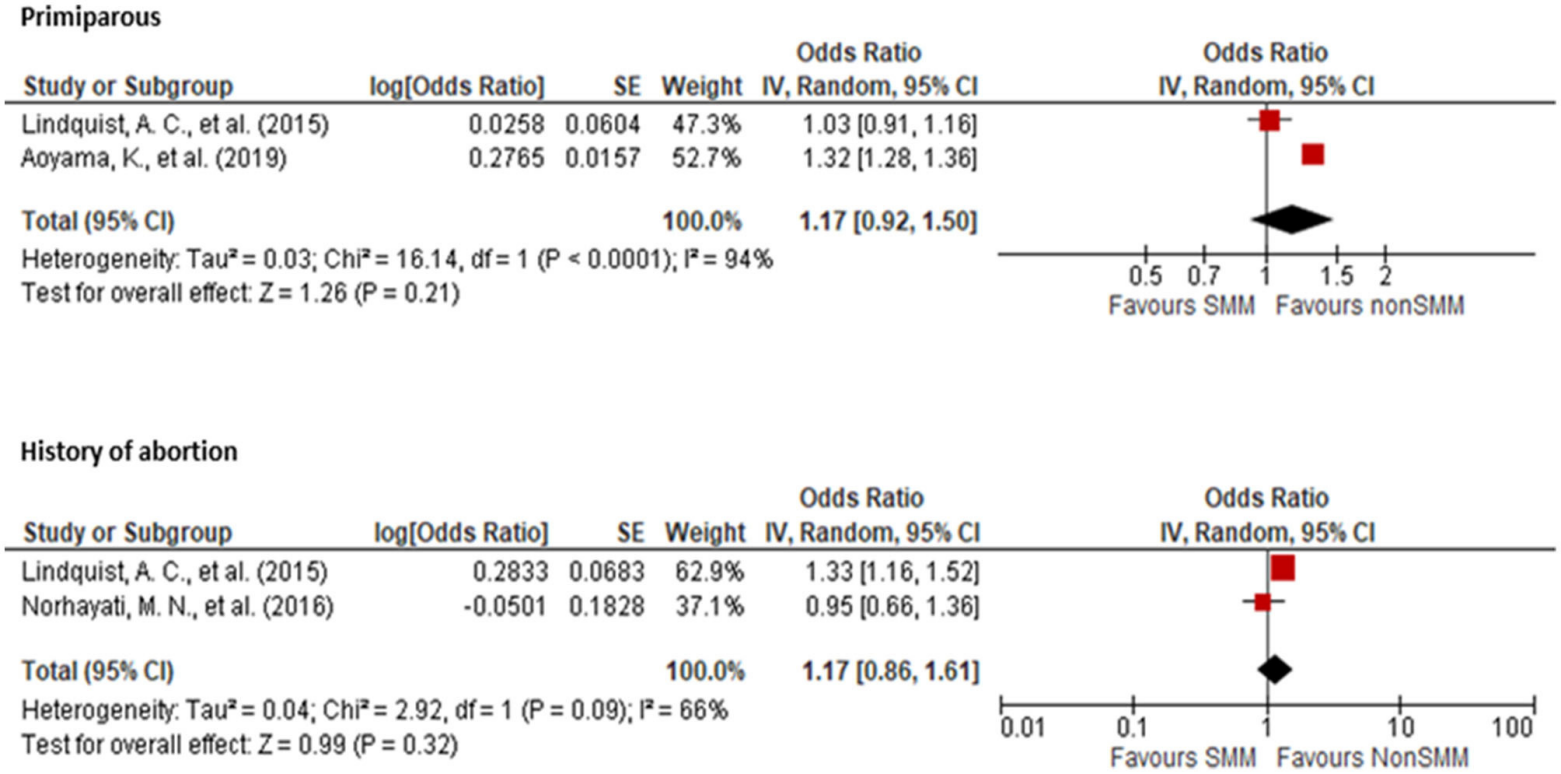
Forest plot showing the association of parity and history of abortion with severe maternal morbidity.

Two studies (19, 25) showed no association between parity and SMM occurrence with an OR of 1.17 for primiparous women (95% CI: 0.92, 1.50) when compared to multiparous women. Likewise, two studies (12, 19) showed no association between history of abortion and SMM occurrence with an OR of 1.17 (95% CI: 0.86, 1.61) (Figure 4) when compared to women without a history of abortion.

**Figure 5:**
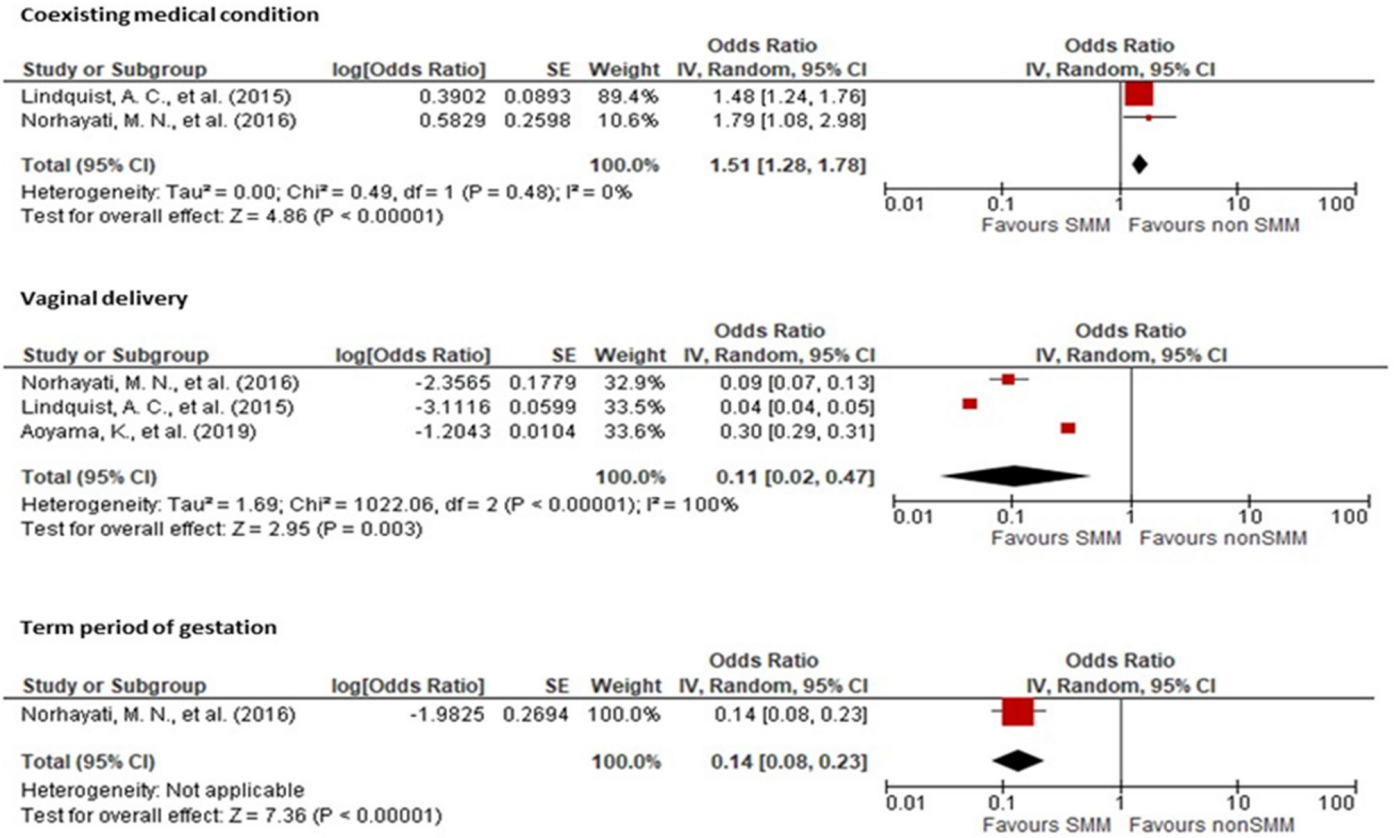
Forest plot showing the association of history of coexisting medical conditions, vaginal delivery, and gestational period with severe maternal morbidity.

Two studies (12, 19) showed a significant association between coexisting medical conditions and SMM with an OR of 1.51 (95% CI: 1.28, 1.78) when compared to mothers without medical conditions. Three studies (12, 19, 25) showed an association between vaginal delivery and SMM with an OR of 0.11 (95% CI: 0.02, 0.47) when compared to cesarean delivery. Lastly, one study showed an association between full-term delivery and SMM with an OR of 0.14 (95% CI: 0.08, 0.23) when compared to pre-term delivery (Figure 5).

### Maternal Near Miss

**Figure 6:**
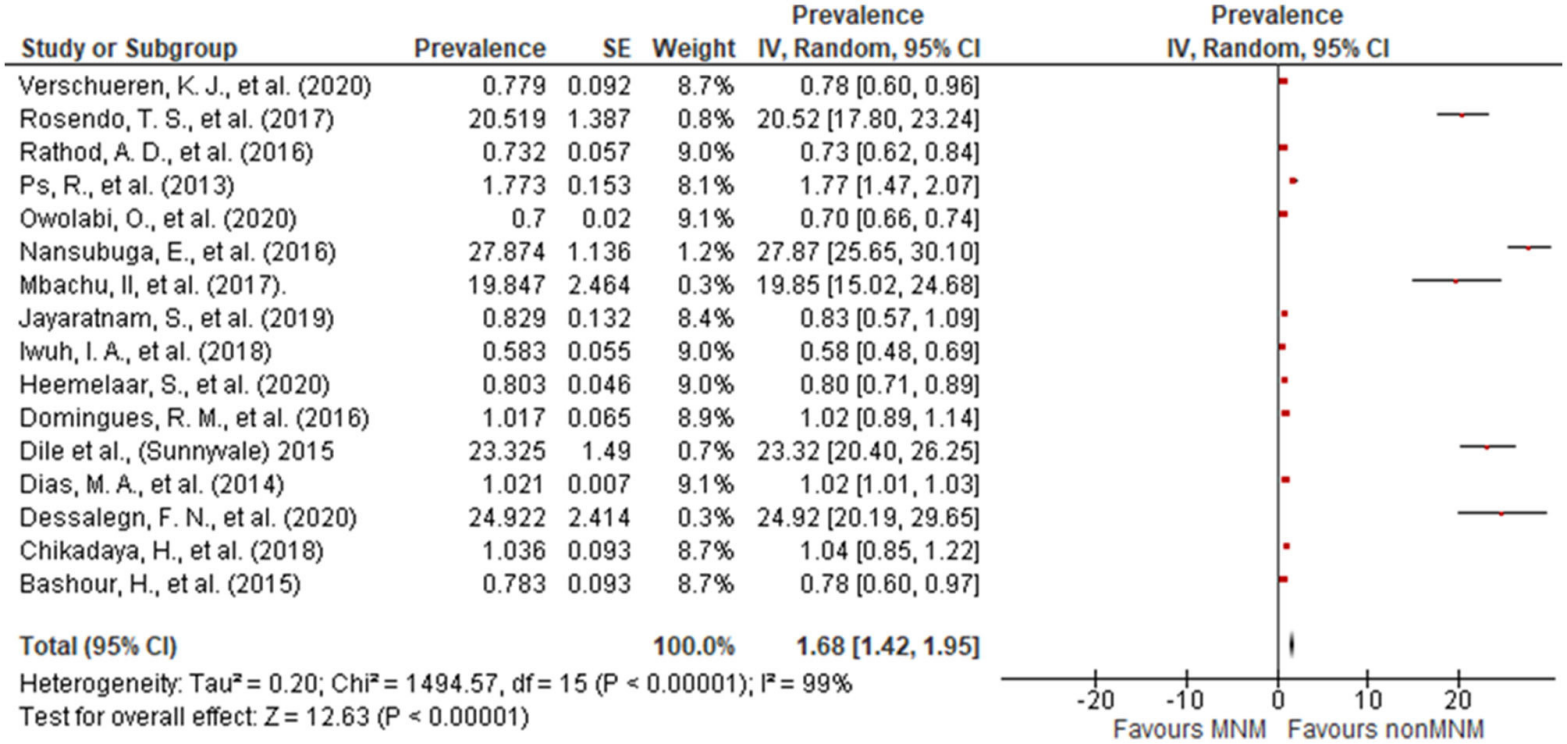
Forest plot depicting the prevalence of maternal near miss.

Sixteen articles were included in the estimation of the pooled prevalence of MNM. A wide variance in the prevalence of MNM was observed. The lowest reported prevalence was 0.58% (33) and the highest was 27.87% (28). The overall pooled prevalence of MNM was 1.68% (95% CI: 1.42, 1.95) (Figure 6).

**Figure 7:**
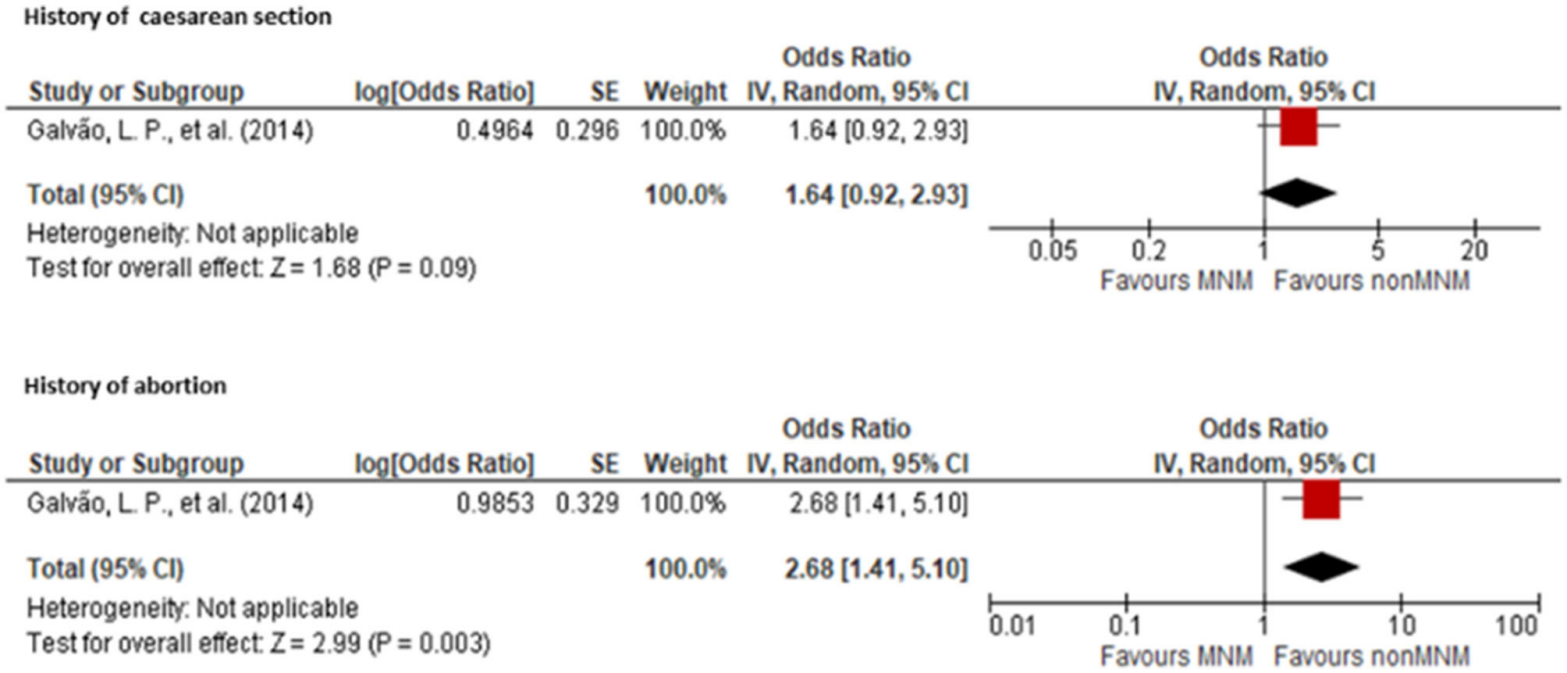
Forest plot showing the association of history of cesarean section and abortion with maternal near miss.

One study (23) reported an association between a history of cesarean section and MNM with an OR of 2.68 (95% CI: 1.41, 5.10) compared to women without a history of cesarean section. Additionally, the same study (23) showed an association between a history of abortion and MNM with an OR of 1.64 (95% CI: 0.92, 2.93) when compared to women with no history of abortion (Figure 7).

## Discussion

In our analysis, the worldwide prevalence of SMM and MNM was 2.45 and 1.68%, respectively. The risk factors of history of cesarean section, advanced maternal age, multiple pregnancies, co-existing medical conditions, and cesarean birth were associated with SMM, while the sole reported risk factor for MNM was a history of cesarean section.

Older maternal age has been associated with a greater likelihood of having pre-existing medical conditions, a higher risk of obstetric complications, maternal morbidity, and an increased risk of progression from SMM to death (42). In our review, it can be concluded that advanced maternal age increases the risk of SMM. Contrary to the results of other reviews, we found that maternal morbidity was higher in mothers at both ends of the age spectrum, such as those of age >35 years and adolescents (43).

According to a review (43), national estimates from vital registration in the United States suggest that adolescents have a lower MMR than women of age >20 years. The same review also reported statistics from the United Kingdom showing that the MMR for women of age under 20 years is slightly higher than that of women of age 20–24 years but lower than that of women of age >25 years. Conversely, data from Australia suggest that the MMR for women of age under 20 years is higher than those of all other age groups apart from women of age >40 years. Evidence from both developing and developed countries also suggests that risks are greater for younger adolescents, with girls of age 15 years or younger having higher mortality rates than older adolescents. In most countries, adolescent births are concentrated among poorer, less educated women, which further compounds their disadvantaged situation by disrupting school attendance and limiting future livelihood opportunities.

In terms of physiological risk, the evidence is somewhat conflicting. Still, some evidence suggests that young women are at increased risk of several direct causes of maternal mortality, such as eclampsia, and indirect causes, such as obstructed labor. A study in Latin America also showed increased risks of hemorrhage and sepsis in young women, particularly those under 16 years of age (43).

According to a report by the Centers for Disease Control and Prevention, the MMR in the United States increased gradually with increasing maternal age and particularly in women older than 35 years, specifically, a 3.5-fold increase in MMR. This increased rate of maternal mortality is mostly attributed to comorbidities and coexisting medical conditions, such as metabolic syndrome, past and current cancer, cardiovascular, renal, and autoimmune diseases, which are more prevalent among older pregnant women. For instance, hypertension and Type II diabetes are more common in older women, and women older than 35 years have a 2- to 4-fold higher risk of having hypertension compared to women aged 30–34 years (44).

Women with a prior cesarean delivery had almost two times the risk (CI: 1.93, 2.23) of SMM compared to those without a prior cesarean delivery. Prior cesarean delivery is known to be associated with preeclampsia, placenta previa, placenta accreta, placental abruption, uterine rupture, postpartum infection, transfusion, and admission to the intensive care unit (14). The risks of placenta previa and morbidly adherent placenta in subsequent pregnancies increased markedly with an increasing number of prior cesarean sections. Compared with those without a history of cesarean section, pregnant women with a history of cesarean section were significantly more likely to experience uterine rupture, morbidly adherent placenta, MNM, severe maternal outcomes, and placenta previa. Uterine rupture during pregnancy and delivery is one of the most devastating obstetric complications, as it frequently results in life-threatening maternal and fetal compromises (45).

According to the 2018 annual report by the National Perinatal Epidemiology Center of Ireland, 45.4% of women who experienced an SMM in 2016 were nulliparous (46). The SMM rate for multiparous women was 6.46 per 1,000, 32% higher than that of primiparous women. However, nulliparous women had the highest SMM of 7.66 per 1,000 births, 56% higher than primiparous women at 4.91 per 1,000 births.

A French study found that the risk for SMM was significantly higher for unplanned cesarean deliveries at all maternal ages. In contrast, the planned delivery mode analysis showed an increased risk of SMM associated with planned cesarean delivery compared with planned vaginal delivery in only women aged 35 years and older (42).

According to a secondary analysis by the WHO Multicountry Survey on Maternal and Newborn Health (WHOMCS), a cross-sectional study performed in 29 countries, it was found that twin pregnancies had a higher risk of contributing to SMM and mortality in all regions (47). Using the new WHO diagnostic criteria for severe maternal conditions, another recent WHOMCS study identified that twin pregnancies doubled the risk of SMM, tripled the risk of MNM, and increased the risk of maternal death 4-fold when compared to singleton pregnancies. The current review found a higher occurrence of preterm deliveries in SMM, which correlates with the underlying etiology of the women's medical conditions.

According to the WHO, maternal deaths have been described as the tip of the iceberg and maternal morbidity as the base (5). For every woman who dies from pregnancy-related reasons, another 20 to 30 women suffer from acute or chronic morbidity, frequently with long-term consequences that impair their ability to function normally. Women's physical, mental, and sexual health, as well as their ability to perform in particular areas (e.g., cognition, mobility, and social involvement), body image, and socioeconomic position, are all affected by these consequences. Maternal morbidity, like maternal mortality, is expected to be the highest in low- and middle-income nations, particularly among the poorest women. There are no proper standardized results on the implications of SMM. However, it is well-known that it can lead to short- and long-term consequences, which are preventable.

The studies in the current review underwent quality assessment, and only good-quality studies with a low risk of bias were included. There were only a few studies that compared women with and without maternal complications, which did not allow us to identify population risk factors. The heterogeneity in the outcomes ranged from low to high, possibly due to the participants' sociodemographic characteristics. However, subgroup analysis was not possible because of the limited number of studies that had substantial heterogeneity. We involved only three databases for searching. However, MEDLINE is the most extensive database for social and medical sciences and the inclusion of 24 papers is strong enough to conclude the outcome. Moreover, we have displayed the cumulative effect estimates in forest plots that consolidate the findings.

## Conclusions

Notably, identifying the risk factors for severe maternal morbidity and maternal near miss is pertinent to prevent maternal death. Quantifying the strength of association of the risk factors and surveillance of the presence of these factors at primary and tertiary care levels helps to identify the interventions required that can be performed during antenatal care and childbirth. Initiatives such as educational programs, campaigns, and early detection of risk factors contributing to SMM and MNM are recommended. Proper follow-up is important to monitor the progression of maternal health during the antenatal and postnatal periods. We recommend additional studies comparing women with and without SMM and MNM using the WHO definition and criteria.

## Data Availability Statement

The original contributions presented in the study are included in the article/Supplementary Material, further inquiries can be directed to the corresponding author/s.

## Author Contributions

NHNH, MNN, and ISB: conceptualization, methodology, and visualization. MNN and NHNH: validation, resources, and data curation. MNN and HRMK: formal analysis and writing of original draft preparation. HRMK: investigation. NHNH, MNN, ISB, and HRMK: writing of review and editing. NHNH: project administration. All authors have read and agreed to the published version of the manuscript.

## Conflict of Interest

The authors declare that the research was conducted in the absence of any commercial or financial relationships that could be construed as a potential conflict of interest.

## Publisher's Note

All claims expressed in this article are solely those of the authors and do not necessarily represent those of their affiliated organizations, or those of the publisher, the editors and the reviewers. Any product that may be evaluated in this article, or claim that may be made by its manufacturer, is not guaranteed or endorsed by the publisher.
