# Supplementary material for: The Prevalence and Risk Factors for Severe Maternal Morbidities: A Systematic Review and Meta-Analysis
Source: Front Med (Lausanne). 2022 Mar 17;9:861028. doi: 10.3389/fmed.2022.861028 (PMC8968119; doi:10.3389/fmed.2022.861028)
Supplement: Supplementary file 1 [file Table_1.DOCX]

**Supplementary file 1. Source of funding**

| No. | Author | Funding |
| --- | --- | --- |
| 1 | Lindquist, A. C., et al. (19) | Victorian Government’s Operational Infrastructure Support Program. ACL was funded by a scholarship from the Rhodes Trust. MK is funded by a National Institute for Health Research (NIHR) Professorship. |
| 2 | Hitti, J., et al. (23) | Washington Department of health Contract HED21138 (Perinatal Regional Network) provided salary support for Ms Walker |
| 3 | Dzakpasu, S., et al. (35) | Canadian Institutes of Health Research (PER‐150902) |
| 4 | Das, I., et al. (36) | Not reported |
| 5 | Galvão, L. P., et al. (20) | FAPITEC/SE/FUNTEC/CNPQ n.12/2009 |
| 6 | Zhang, T., et al. (37) | Science and Technology Department of Hebei Province, China (2019YX013A, 18397779D) |
| 7 | Norhayati, M. N., et al. (21) | Short Term Research Grant (304/PPSP/61312141) from Universiti Sains Malaysia |
| 8 | Aoyama, K., et al. (22) | Canadian Institutes for Health Research Transitional Open Operating Grant (342397) |
| 9 | Bashour, H., et al. (38) | WHO Alliance grant (Implementation Research Protocol ID A65770) |
| 10 | Dessalegn, F. N., et al. (39) | Hawassa University College of Medicine and Health Science |
| 11 | Nansubuga, E., et al. (25) | Partially funded by an African Doctoral Dissertation Research Fellowship award offered by the African Population and Health Research Center in partnership with the International Development Research Centre; Makerere University, Uganda; and North West University, South Africa |
| 12 | Rosendo, T. S., et al. (40) | Conselho Nacional de Desenvolvimento Científico e Tecnológico (CNPq), under process number 477496/2011–3 |
| 13 | Chikadaya, H., et al. (41) | None |
| 14 | Rathod, A. D., et al. (42) | Not reported |
| 15 | Verschueren, K. J., et al. (43) | None |
| 16 | Iwuh, I. A., et al. (24) | None |
| 17 | Dias, M. A., et al. (44) | National Council for Scientific and Technilogical Development (CNPq); Science and Tecnology Department, Secretariat of Science, Tecnology, and Strategic Inputs, Brazilian Ministry of Health; National School of Public Health, Oswaldo Cruz Foundation (INOVA Project); and Foundation for supporting Research in the State of Rio de Janeiro (Faperj) |
| 18 | Domingues, R. M., et al. (45) | National Council of Technological and Scientific Development (CNPq); the National School of Public Health, Oswaldo Cruz Foundation (INOVA Project); and the Research Funding Agency of the State of Rio de Janeiro (FAPERJ) |
| 19 | Heemelaar, S., et al. (46) | The Ministry of Health and Social Services of Namibia and the University of Namibia contributed to the travel costs of members of the National Maternal Death Review Committee to visit participating facilities. |
| 20 | Mbachu, II, et al. (47) | None |
| 21 | Ps, R., et al. (48) | Not reported |
| 22 | Dile, M.,et al. (49) | Not reported |
| 23 | Jayaratnam, S., et al. (50) | Not reported |
| 24 | Owolabi, O., et al. (51) | Guttmacher Institute by UK Aid from the UK Government and the Dutch Ministry of Foreign Affairs and to APHRC through generous support from the William and Flora Hewlett Foundation (Grant # 2015-3063) as well as the Segal Family Foundation |

Note: The funding source had no role in the study design, data collection, analysis or interpretation or manuscript preparation.
